# A shift in Clinical Learning Modalities—Telemedicine Training in UK Undergraduate Medical Students

**DOI:** 10.1007/s40670-020-01200-y

**Published:** 2021-01-09

**Authors:** Celion Tang, Anjeevan Kaur Klair, Amir Hussain

**Affiliations:** 1 Faculty of Biology, Medicine and Health, University of Manchester, Oxford Road, Manchester, M13 9PL UK; 2 Manchester, UK

Dear editor,

As final year medical students having recently returned to clinical placements, we found Caplan et al.’s [1] article highly engaging. We agree that the use of simulation mannikins and online lectures are good teaching alternatives outside clinical settings. However, as echoed by recently graduated junior doctors [2], patient encounters are irreplaceable teaching opportunities. As mentioned by the author, such patient interactions train both diagnostic and communication skills. Replacing such teachings with inanimate mannikins neglects the fundamental communication training aspect of patient-encounters and therefore is not an entirely adequate teaching solution. Furthermore, NHS is shifting to telemedicine (online, telephone, or video call consultations) to minimize the risk of COVID-19 transmissions, altering the dynamics of clinical interactions [3, 4]. We propose to incorporate telehealth training into our curriculum to both compensate for the lack of communication training from using mannikins and prepare the next wave of doctors for this emerging patient-encounter platform.

Telemedicine, although effective and safe, has its challenges. It requires specific skillsets to overcome any technical or technological difficulties, at the same time building a good rapport with patients distantly. Clinicians without prior exposure to telehealth found it challenging to gather detailed history and establish appropriate working diagnoses. Certain communication skills, especially clear pronunciations and minimal body gestures are keys in assessing patients remotely [5]. Additionally, students were more comfortable utilizing telemedicine after taught about the laws and ethics revolving telehealth applications [6].

In our own experiences, we found the demonstration of active listening with our body languages and eye contact was significantly limited, whereas verbal encouragements and summarizing were useful to build rapport remotely. Adapting to this digital form of patient interaction poses challenges especially considering undergraduate training has been primarily based on face-to-face encounters. As such, we believe that appropriate telehealth training is essential for medical students to familiarize and overcome the challenges in telecommunication discussed above. Although remote, it offers an alternative answer for face-to-face patient encounter communication training, not provided with the use of mannikins and without a concern for personal protective equipment (PPE).

In conclusion, this pandemic has called for a shift from the conventional delivery of clinical teachings. Some international medical schools had already implemented telemedicine training in their undergraduate curriculum [6]. Collaborative work between UK and overseas medical institutions would be beneficial to outline an effective telehealth training plan for UK medical students, that is in accordance to the available national guidelines on telehealth applications [3, 4, 7].

## Data Availability

Not applicable
